# Ni, Co, Zn, and Cu metal-organic framework-based nanomaterials for electrochemical reduction of CO_2_: A review

**DOI:** 10.3762/bjnano.14.74

**Published:** 2023-08-31

**Authors:** Ha Huu Do, Hai Bang Truong

**Affiliations:** 1 VKTech Research Center, NTT Hi-Tech Institute, Nguyen Tat Thanh University, Ho Chi Minh City, 700000, Vietnam; 2 Optical Materials Research Group, Science and Technology Advanced Institute, Van Lang University, Ho Chi Minh City, Vietnam; 3 Faculty of Applied Technology, School of Technology, Van Lang University, Ho Chi Minh City, Vietnam

**Keywords:** carbon capture, CO_2_ reduction, electrocatalysis, metal-organic frameworks, nanomaterials

## Abstract

The combustion of fossil fuels has resulted in the amplification of the greenhouse effect, primarily through the release of a substantial quantity of carbon dioxide into the atmosphere. The imperative pursuit of converting CO_2_ into valuable chemicals through electrochemical techniques has garnered significant attention. Metal-organic frameworks (MOFs) have occured as highly prospective materials for the reduction of CO_2_, owing to their exceptional attributes including extensive surface area, customizable architectures, pronounced porosity, abundant active sites, and well-distributed metallic nodes. This article commences by elucidating the mechanistic aspects of CO_2_ reduction, followed by a comprehensive exploration of diverse materials encompassing MOFs based on nickel, cobalt, zinc, and copper for efficient CO_2_ conversion. Finally, a meticulous discourse encompasses the challenges encountered and the prospects envisioned for the advancement of MOF-based nanomaterials in the realm of electrochemical reduction of CO_2_.

## Introduction

The emission of carbon dioxide resulting from the utilization of fossil fuels has been identified as a primary cause of the greenhouse effect, ultimately contributing to the severity of climate change [1]. To mitigate these detrimental consequences, numerous strategies have been implemented to address the issue of CO_2_ emissions. Among these, the carbon capture and storage (CCS) technique plays a crucial role in curtailing the release of CO_2_ into the air. By capturing and containing approximately 90% of the CO_2_ gas generated through the combustion of conventional fuels utilized for human energy consumption [2–3], this method proves instrumental in abating the pollution caused by CO_2_. Nevertheless, CO_2_ storage and transportation are expensive, necessitating the development of efficient adsorbents [3]. An auspicious avenue to tackle these challenges is the conversion of CO_2_ into valuable compounds through electrochemical reduction. The electrocatalytic process for CO_2_ reduction reactions (CO_2_RR) encounters a persistent obstacle in the activation of CO_2_ [4]. The formation of CO_2_^•−^ necessitates a high thermodynamic potential of −1.90 V vs the standard hydrogen electrode. Subsequently, multiple electron transfers occur, leading to the generation of diverse products such as ethanol, methanol, and methane [5–7]. Therefore, to reduce the activation energy and to improve selectivity, the meticulous consideration of catalysts becomes imperative [8–16]. The first work on electrocatalytic CO_2_ reduction was published in 1870 using a Zn material to produce HCOOH [17–18]. Subsequent investigations have yielded numerous studies focusing on the development of electrocatalysts for CO_2_RR. In 1994, Hori et al. highlighted that the selectivity of products exhibited considerable variation depending on the elemental composition of pure metal catalysts [19]. Notably, Au, Ag, and Zn catalysts exhibit preferential CO generation, while Sn, In, and Pb catalysts prove effective in producing formate ions (HCOO^−^) [20].

Metal-organic frameworks (MOFs) are established from metal ions and organic linkers, and have been identified as prospective materials for CO_2_RR [21]. Therefore, a multitude of MOFs structures have been explored in experimental studies [22–23], exhibiting diverse applications such as gas storage [24], electrocatalysis [25–27], glucose sensing [28–30], and biomedical [31] applications. These materials are distinguished by their exceptional attributes, including a substantial specific surface area, pronounced porosity, and modifiable chemical structures [32]. Within the catalytic domain, MOFs demonstrate catalytic activity stemming from both their metal sites and organic components. Furthermore, their catalytic properties can be readily modified through functionalization. For instance, Fu et al. grafted –NH_2_ groups onto MIL-125(Ti) material to enhance CO_2_RR for the production of HCOO^−^ [33]. The outcome indicated that NH_2_-MIL-125(Ti) indicated superior catalytic activity compared to MIL-125(Ti). Notably, MOF-210 has established a remarkable record in CO_2_ adsorption among all porous materials, boasting an adsorption capacity of 2870 mg·g^−1^ [34]. Such properties facilitate favorable interactions between CO_2_ molecules and catalytic sites within MOFs, thereby enhancing the CO_2_RR process. Besides, MOFs could be used as ideal precursors for the controlled dispersion of metal nanoparticles within organic frameworks, either through operational conditions or via the pyrolysis technique, thereby promoting efficient CO_2_ reduction [35–36]. The augmentation of MOF properties can be accomplished by converting pristine MOFs into nanoscale materials. A diverse array of MOF nanomaterials has been reported, encompassing single-atom nanocatalysts (SACs), hetero-atom-doped nanomaterials, and MOF nanofiber-based aerogels, among others, as highlighted by Behera et al. in 2022 [37]. These derived nanomaterials showcase enhanced stability, favorable morphologies, advanced functionalities, and precisely controlled textural characteristics in comparison to their original MOF counterparts.

Herein, we provide a comprehensive overview of the latest literature pertaining to the implementation of MOF-based nanomaterials for the electrochemical conversion of CO_2_. First, the reaction pathway of the CO_2_ reduction is described for the production of different chemicals. Then, various structures, including Ni-, Co-, Zn-, and Cu-based MOFs for electrochemical CO_2_RR are presented. Finally, we present the potential pathways and current problems in progressing MOF-based nanomaterials for CO_2_ conversion.

## Review

### Mechanism of CO_2_RR

**Figure 1:**
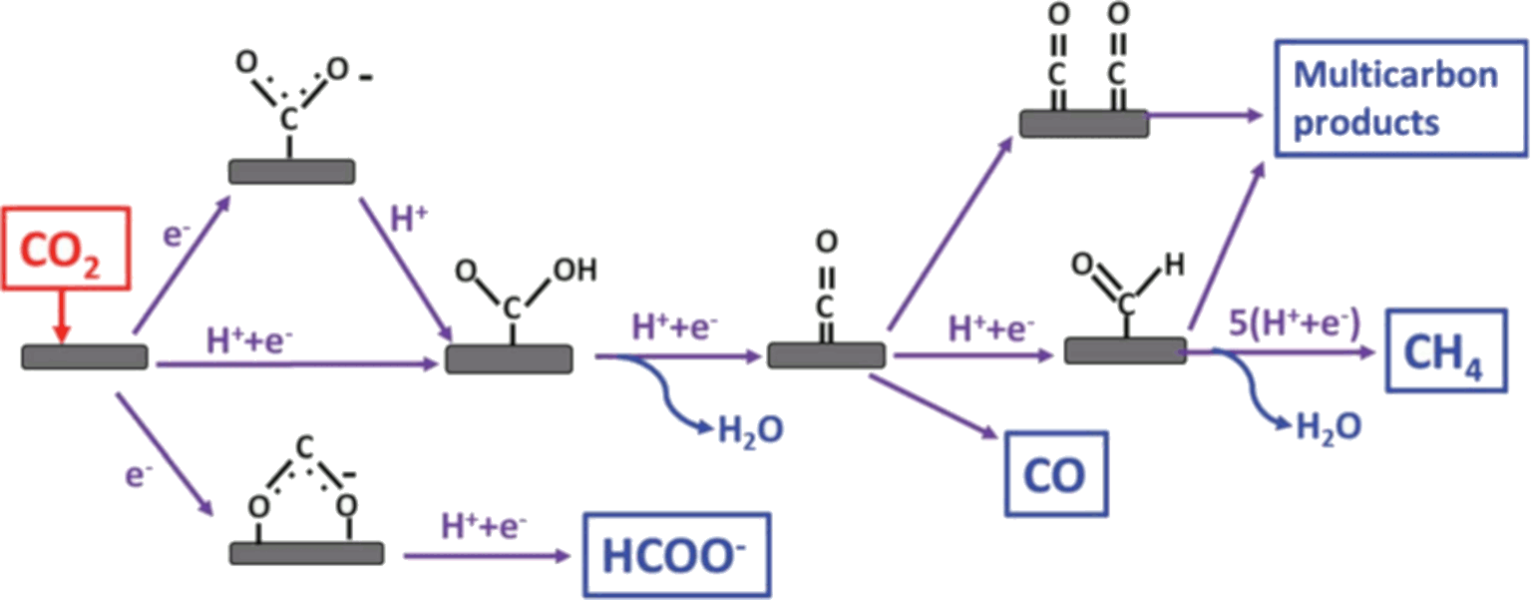
Schematic illustration of CO_2_RR for various chemicals production. Republished with permission of The Royal Society of Chemistry, from [38], (“CO_2_ electrochemical reduction on metal-organic framework catalysts: current status and future directions” by D. Narváez-Celada and A. S. Varela, J. Mater. Chem. A, Vol. 10, Issue 11, © 2022); permission conveyed through Copyright Clearance Center, Inc. This content is not subject to CC BY 4.0.”.

The process of CO_2_ reduction consists of three steps. First, the CO_2_ molecules are adsorbed on the active sites of catalysts. Second, charge transfer processes take place to create intermediates such as *CHO, *CO, and *COO. The process could include many electrons attending in the electrochemical reaction, and orientate the formed products. Finally, these species are desorbed from the active sites of electrocatalysts to generate various products, as shown in Figure 1. In addition to the properties of the catalyst material, other parameters, such as potential, pH, solvent, and temperature, also determine the formation of desired products.

### MOFs nanomaterials for electrocatalytic reduction of CO_2_

#### Ni-based MOFs nanomaterials

**Figure 2:**
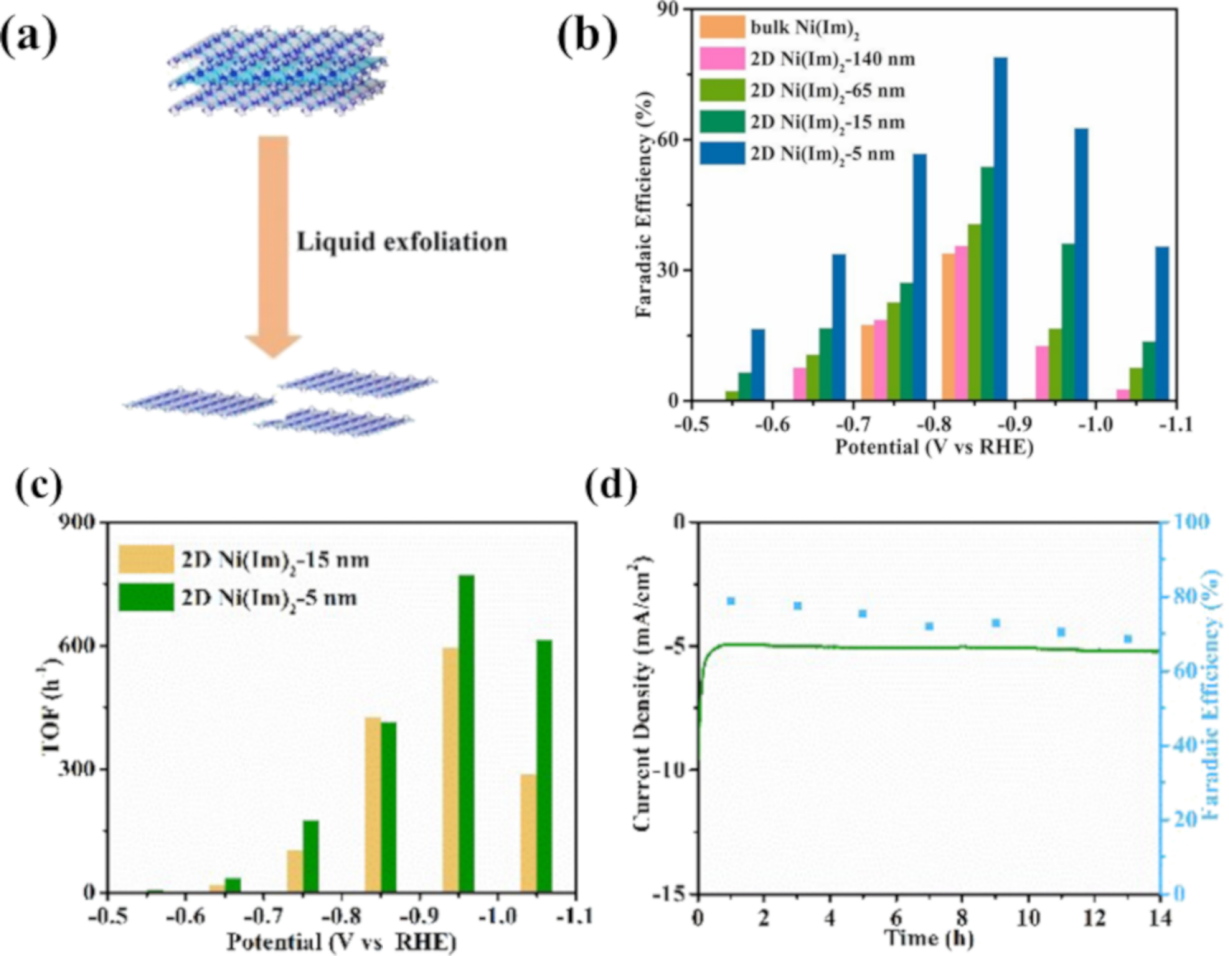
(a) Graphic representation of the synthesis of 2D Ni(Im)_2_, (b) Faradaic efficiency of 2D Ni(Im)_2_ with various thicknesses, (c) TOF of 2D Ni(Im)_2_-5 nm and 2D Ni(Im)_2_-15 nm, (d) durability test and FE of 2D Ni(Im)_2_-5 nm at −0.85 V vs RHE for 14 h. Figure 2 was adapted with permission of The Royal Society of Chemistry, from [39], (“Ultrathin 2D nickel zeolitic imidazolate framework nanosheets for electrocatalytic reduction of CO_2_” by J.-X. Wu et al., Chemical Communications, Vol. 55, Issue 77, © 2019); permission conveyed through Copyright Clearance Center, Inc. This content is not subject to CC BY 4.0.

Two-dimensional (2D) MOFs represent a novel addition to the family of 2D materials. Particularly, 2D MOF nanolayers with several outstanding characteristics, such as high surface area and abundant exposed active sites, have been studied for CO_2_RR. As a case in point, Wu et al. prepared 2D Ni-based zeolitic imidazole framework (ZIF) nanosheets as efficient material for electrochemical CO_2_ conversion [39]. 2D Ni(Im)_2_ materials with various thicknesses were fabricated through varying centrifugation speeds (Figure 2a). The outcome revealed that the optimal sample, possessing a thickness of 5 nm, yielded the highest performance of CO production (FE_CO_ = 78.8% at −0.85 V vs RHE), compared to its bulk counterpart with a value of 33.7% (Figure 2b). The optimal sample also showed a high turnover frequency (TOF) and outstanding stability after a testing period of 14 h (Figure 2c,d). The high catalytic activity can be ascribed to the enhanced number of active sites achieved through the transition from the bulk state to the nanomaterial form. The optimal condition for the fabrication of 2D Ni(Im)_2_ was determined to be the utilization of 5 mL of NH_4_OH.

#### Co-based MOFs nanomaterials

**Figure 3:**
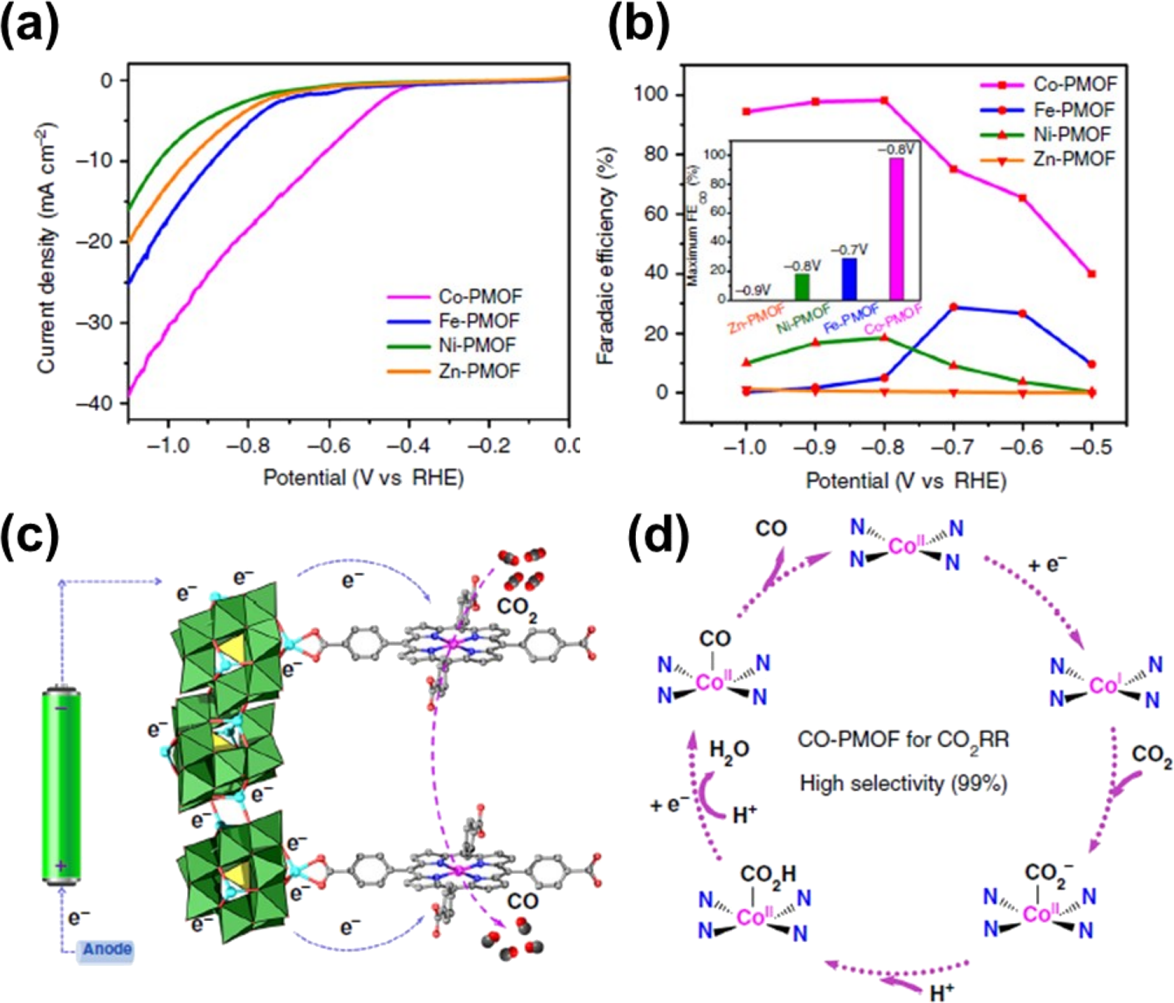
(a) Polarization curves of various M-PMOFs (M = Co, Ni, Fe, Zn) for CO formation, (b) Faradaic efficiency for CO product on different materials, (c, d) proposed reaction pathway for the electrochemical CO_2_ conversion with Co-PMOF. Figure 3 was adapted from [41]; (© 2018 Y.-R. Wang et al., published by Springer Nature, distributed under the terms of the Creative Commons Attribution 4.0 International License (CCBY 4.0), http://creativecommons.org/licenses/by/4.0/).

Cobalt materials provide a diversity of reduction–oxidation states and are, thus, considered potential candidates in electrocatalysis. Co-related MOFs have been extensively investigated for their applicability in CO_2_ conversion processes. Wang et al. introduced an interesting work based on four distinct structures, including Co-PMOF, Ni-PMOF, Fe-PMOF, and Zn-PMOF (P: polyoxometalate) for CO_2_ conversion. Co-PMOF displayed the highest catalytic activity for CO_2_RR among the investigated MOFs, as illustrated in Figure 3a,b. Moreover, this catalyst also showed remarkable durability, with the current density remaining stable after 35 h of testing. To gain insights into the reaction pathway and to provide explanations for the observed outcome, the research team employed computational science techniques. Density functional theory (DFT) calculations implied that Co-PMOF possessed the lowest total free energy leading to its superiority as a catalyst for CO_2_RR. The author postulated that Co(II) is converted into Co(I), which acts as a redox center for the reduction of CO_2_ into CO (Figure 3c,d). Because of their poor conductivity, Co-MOFs are typically grown on conductive templates, such as fluorine-doped tin oxide (FTO), carbon cloth, and carbon pastes, which serve as cathodes for CO_2_RR. To illustrate, Kornienko et al. deposited a Co-based MOF material onto an FTO substrate as a working electrode for CO_2_ conversion [40]. This material exhibited good performance in CO generation, achieving a faradaic efficiency (FE) of 76% (at −0.7 V vs RHE). The authors attributed the active center for CO_2_ conversion to Co(I) species generated through the reduction of Co(II).

#### Zn-based MOFs nanomaterials

**Figure 4:**
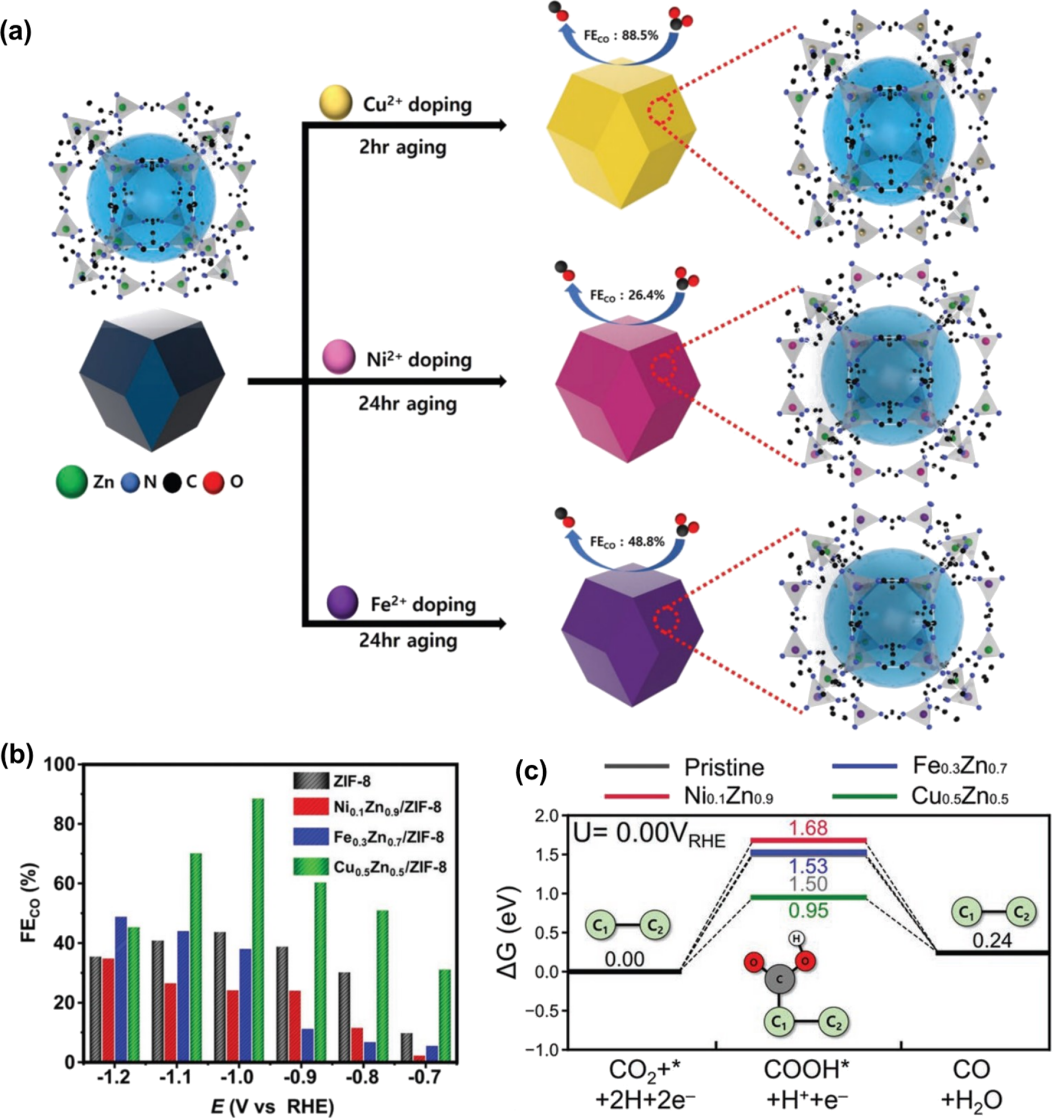
(a) A graphic representation of the preparation of M_z_Zn_y_/ZIF-8, (b) Faradaic efficiency for the CO production using different materials, (c) diagram of free energy for CO_2_RR. Figure 4 was adapted from [44], J. H. Cho et al., “Transition Metal Ion Doping on ZIF-8 Enhances the Electrochemical CO_2_ Reduction Reaction”, Advanced Materials, with permission from John Wiley and Sons. Copyright © 2022 Wiley-VCH GmbH. This content is not subject to CC BY 4.0.

Zinc (Zn) metal-based electrocatalysts have been identified as outstanding candidates for CO_2_ conversion into CO because of their low cost, nontoxic nature, and high efficiency. Therefore, considerable interest has been directed towards exploring the potential of Zn-based MOFs in CO_2_RR applications. Wang and co-workers successfully prepared ZIF-8 from different metal salts for CO_2_RR [42]. ZIF-8 derived from ZnSO_4_ yielded the best performance for the electrochemical reduction of CO_2_ into CO, with an FE of 65%. The research group also revealed that the Zn^2+^ species operate as active sites in the catalytic process. Another investigation shed light on the significance of organic ligands within Zn-based MOF architectures for CO_2_RR. Jiang et al. prepared four Zn-based ZIFs, including ZIF-7, ZIF-108, ZIF-8, and SIM-1, employing various ligands while utilizing the same Zn-containing salt [43]. These architectures were evaluated under identical conditions to determine the role of ligands in CO_2_ conversion. The ZIF-8 variant with the 2-methylimidazole ligand exhibited the highest activity for CO_2_RR to carbon monoxide (FE = 81% at −1.1 V vs RHE). This can be explained by the fact that ZIF-8 has the smallest adsorption energy of hydrogen, facilitating the desired CO_2_RR process. The outcomes of this study serve as a foundation for the exploration of transition metal ion doping in ZIF-8, aiming to enhance the performance of CO_2_ conversion, as recently reported (Figure 4a). Cho et al. revealed that Cu-doped ZIF-8 exhibited the highest catalytic activity, surpassing both Fe- and Ni-doped ZIF-8 [44]. Specifically, Cu_0.5_Zn_0.5_/ZIF-8 yielded a large FE_CO_ of 88.5% at −1.0 V (RHE), whereas the values were 48.8% and 34.7% for Fe-doped ZIF-8 and Ni-doped ZIF-8 at −1.2 V (RHE), respectively (Figure 4b). These results were confirmed by theoretical calculations, which indicated the lowest COOH adsorption energy of Cu-doped ZIF-8 (Figure 4c).

#### Cu-based MOF nanomaterials

**Figure 5:**
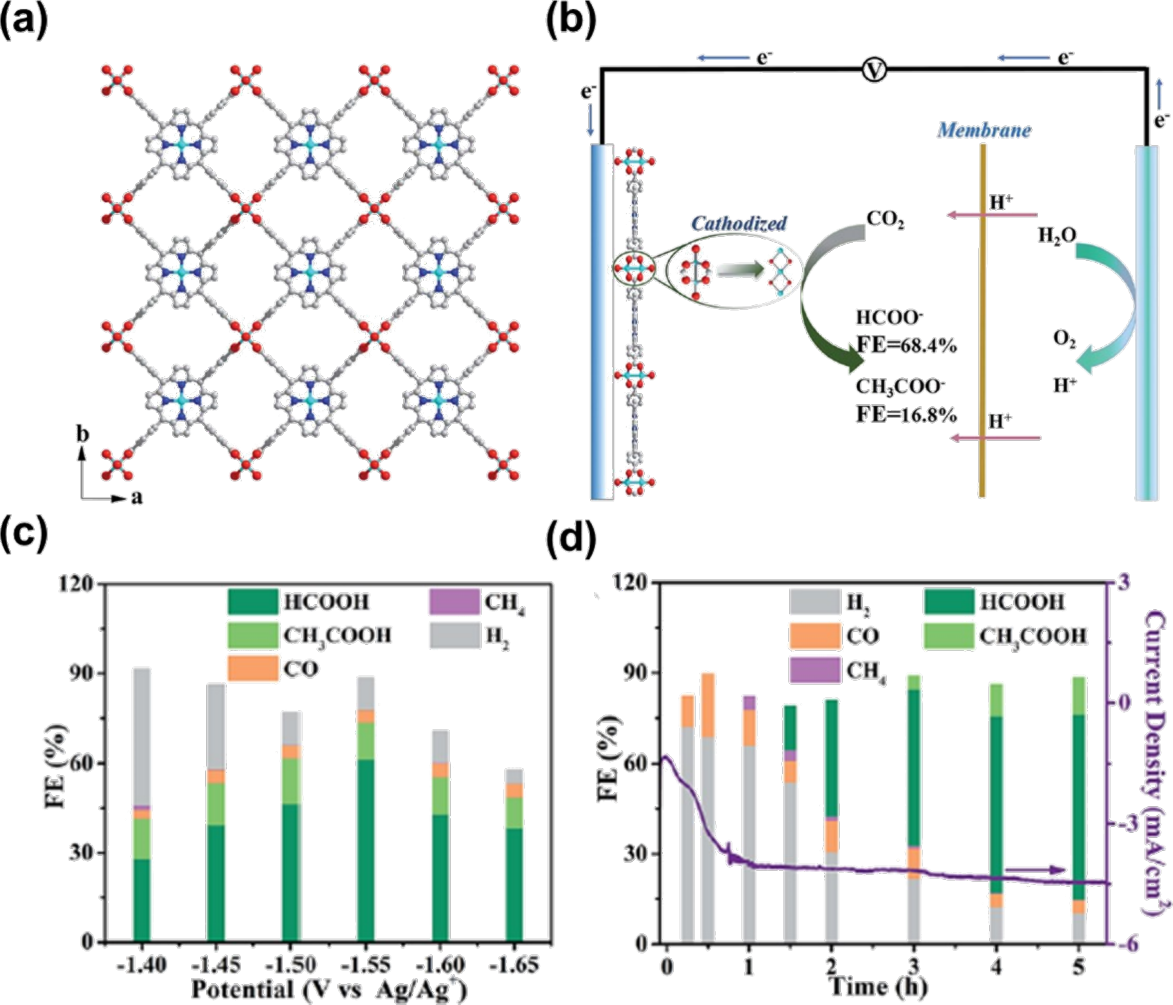
(a) Crystal architecture of 2D Cu_2_(CuTCPP) nanosheets, (b) graphic illustration of the electrochemical CO_2_ reduction, (c) Faradaic efficiency of catalysts at various potentials, (d) Faradaic efficiency of catalysts as functions of the time. Figure 5 was adapted from [49]. (“Cathodized copper porphyrin metal–organic framework nanosheets for selective formate and acetate production from CO_2_ electroreduction”, © 2019 J.-X. Wu et al., published by The Royal Society of Chemistry, distributed under the terms of the Creative Commons Attribution-Non Commercial 3.0 Unported Licence, https://creativecommons.org/licenses/by-nc/3.0/). This content is not subject to CC BY 4.0.

Cu-based MOFs are high-potential materials for the electrochemical CO_2_ reduction because of their cost-effectiveness, nontoxicity, and diversity of active sites. Hinogami et al. reported that a copper rubeanate MOF has a higher catalytic activity than Cu metal for the CO_2_ conversion into formic acid, primarily because of the weak adsorption of CO_2_ on the MOF surface [45]. Solvents also play a vital role in CO_2_ reduction, as highlighted in Kumar’s study [46], where the dimethylformamide solvent supplied protons for HCOOH formation with high purity. Albo et al. assessed the catalytic activity of different Cu-MOFs for CO_2_ reduction [47]. The study revealed that HKUST-1 showed the highest performance with a FE of 15.9% at a current density of 10 mA·cm^−2^ for methanol and ethanol formation. In particular, FE_(methanol)_ is 5.6% and FE_(ethanol)_ is 10.3%. This result can be explained by two reasons. On the one hand, HKUST-1 contains open metal sites (Cu^2+^), which are not hindered by surrounding linkers, facilitating interaction with intermediates and, thus, increasing CO_2_ reduction. On the other hand, the largest surface area also partially contributes to improving the performance of CO_2_ reduction. Later, the authors improved the CO_2_ reduction performance by mixing HKUST-1 with a Bi-based MOF (CAU-17) [48]. The optimized sample demonstrated a considerably elevated FE for alcohol production, reaching 36.9%. This enhancement was attributed to the synergistic effects between Cu- and Bi-MOFs, which played a pivotal role in promoting interactions between active species and transition states. Notably, Bi centers in CAU-17 are the main active sites in the generation of HCOO–, after which these intermediates would move to open Cu metal sites (HKUST-1), thus improving activity. In an interesting study by Wu et al., Cu-based MOF nanosheets were utilized for CO_2_ reduction to formate and acetate (Figure 5a,b) [49]. The authors observed that Cu^2+^ nodes underwent conversion to copper oxides under operational conditions. The presence of these species, along with a porphyrin–Cu(II) complex, resulted in an enhancement of CO_2_ reduction. As a result, this material exhibited a substantial FE of 68.4% for formate generation at a voltage of −1.55 V (Figure 5c,d). However, the performance decreased after 5 h of testing, attributed to a restructuring of the Cu-based MOF. In addition, methane and ethylene were also considered as useful compounds in specific applications. However, the utilization of MOFs as electrocatalysts for the conversion of CO_2_ into hydrocarbons remains relatively limited. A recent study by Yang et al. presented a potential MOF for the reduction of CO_2_ to methane and ethylene [36]. Cu nanoparticles were created during when the Cu^II^/ade-MOFs reconstructed and act as active centers for CO_2_ reduction to CH_4_ and C_2_H_4_.

## Conclusion and Outlook

MOFs were recognized as promising nanomaterials for the transformation of CO_2_ into valuable products through electrochemical processes. This interest arises from their advantageous properties, including high surface area, customizable morphological structures, well-defined metal sites, facile modification, and compositional diversity. Overall, the catalytic generation of diverse products from the electrochemical reduction of CO_2_ is governed by the inherent characteristics of metal sites and organic linkers within the MOF structure. Notably, MOF nanomaterials based on Zn, Co, and Ni have demonstrated potential for CO_2_ reduction to CO, whereas Cu-related MOFs are favorable for the conversion of CO_2_ to formate, formic acid, alcohol, and hydrocarbons. Numerous studies have proposed reaction mechanisms based on the calculation of Gibbs energy for intermediate species, providing insights into the underlying processes involved in CO_2_ electrocatalysis. However, they also encounter some difficulties in the field of CO_2_ reduction. In particular, the low conductivity of MOFs hampers electron transport, leading to sluggish electrochemical reaction kinetics. To alleviate this problem, highly conductive materials such as graphene, and carbon nanotubes were combined with MOFs to improve overall conductivity. Additionally, the usage of pristine MOFs as sacrificial agents to create metal or metal compounds embedded in carbon matrices was considered a potential direction in CO_2_RR application. Systematic studies should be conducted to control morphological structure and composition by changing reaction conditions (time, temperature, and pressure) when converting individual MOFs into MOF-derived carbon-support nanomaterials. Another issue is the durability of the working electrodes. Many studies have employed drop casting and the use of binders to affix MOFs onto the substrate for electrode fabrication. This approach presents drawbacks such as reduced accessibility to active sites and unstable MOFs/substrate interfaces. Therefore, further studies are required to develop binder-free electrodes by in situ synthesis of MOFs on conductive substrates, such as nickel foam, copper foil, and carbon cloth, to overcome the aforementioned limitations and advancing the field of CO_2_RR.
